# COVID-19 outbreak and genomic investigation in an inpatient behavioral health unit

**DOI:** 10.1017/ash.2024.40

**Published:** 2024-04-29

**Authors:** Estefany Rios-Guzman, Alina G. Stancovici, Lacy M. Simons, Grace Barajas, Katia Glenn, Rachel T. Weber, Egon A. Ozer, Ramon Lorenzo-Redondo, Judd F. Hultquist, Maureen K. Bolon

**Affiliations:** 1 Division of Infectious Diseases, Northwestern University Feinberg School of Medicine, Chicago, IL, USA; 2 Center for Pathogen Genomics and Microbial Evolution, Northwestern University Havey Institute for Global Health, Chicago, IL, USA; 3 Department of Healthcare Epidemiology and Infection Prevention, Northwestern Memorial Hospital, Chicago, IL, USA

## Abstract

**Background:**

Inpatient behavioral health units (BHUs) had unique challenges in implementing interventions to mitigate coronavirus disease 2019 (COVID-19) transmission, in part due to socialization in BHU settings. The objective of this study was to identify the transmission routes and the efficacy of the mitigation strategies employed during a COVID-19 outbreak in an inpatient BHU during the Omicron surge from December 2021 to January 2022.

**Methods:**

An outbreak investigation was performed after identifying 2 COVID-19-positive BHU inpatients on December 16 and 20, 2021. Mitigation measures involved weekly point prevalence testing for all inpatients, healthcare workers (HCWs), and staff, followed by infection prevention mitigation measures and molecular surveillance. Whole-genome sequencing on a subset of COVID-19-positive individuals was performed to identify the outbreak source. Finally, an outbreak control sustainability plan was formulated for future BHU outbreak resurgences.

**Results:**

We identified 35 HCWs and 8 inpatients who tested positive in the BHU between December 16, 2021, and January 17, 2022. We generated severe acute respiratory coronavirus virus 2 (SARS-CoV-2) genomes from 15 HCWs and all inpatients. Phylogenetic analyses revealed 3 distinct but genetically related clusters: (1) an HCW and inpatient outbreak likely initiated by staff, (2) an HCW and inpatient outbreak likely initiated by an inpatient visitor, and (3) an HCW-only cluster initiated by staff.

**Conclusions:**

Distinct transmission clusters are consistent with multiple, independent SARS-CoV-2 introductions with further inpatient transmission occurring in communal settings. The implemented outbreak control plan comprised of enhanced personal protective equipment requirements, limited socialization, and molecular surveillance likely minimized disruptions to patient care as a model for future pandemics.

## Introduction

The coronavirus disease 2019 (COVID-19) pandemic placed a massive burden on healthcare systems, prompting changes to normal standard-of-care practices.^1^ To circumvent hospital-acquired severe acute respiratory coronavirus virus 2 (SARS-CoV-2) infections, clinical settings adopted COVID-19-related mitigation strategies applicable to patients, visitors, staff, and healthcare workers (HCWs) that included mandatory masking and social distancing practices.^2,3^ Although these measures were broadly effective at preventing nosocomial SARS-CoV-2 infections, specialized care settings, such as inpatient psychiatric units, continued to experience outbreaks throughout the first year of the pandemic.^4–8^

Inpatient psychiatric treatment units, otherwise referred to as behavioral health units (BHUs), commonly have open floor layouts and employ group-oriented treatments (ie, milieu therapies), which could contribute to nosocomial outbreaks of infection.^9^ Quarantine is not an option for most BHU patients due to isolation limitations^10^ and promotion of socialization.^11^ Targeted use of personal protective equipment (PPE) can likewise lead to stigmatization and a sense of violation of the patient’s right to privacy, which can exacerbate psychiatric symptoms both in the patient and their therapeutic community.^12^ Although universal masking was broadly adopted as a mitigation strategy in healthcare settings, including BHUs, adherence was difficult to monitor and enforce. As patient–patient and patient–HCW interaction remained integral to patient care, an increased prevalence of SARS-CoV-2 transmission within BHU settings in comparison to other medical units was anticipated.^13^

The SARS-CoV-2 Omicron variant that emerged in southern Africa in late 2021 quickly spread around the globe, displacing the previously dominant Delta variant on account of its higher transmissibility, shorter incubation period, and enhanced immune escape that enabled higher rates of reinfection and vaccine escape.^14^ The magnitude of the Omicron surge and its unique properties resulted in a broad re-evaluation of infection control procedures in late December 2021.^15^ In 1 psychiatric hospital alone, 62% of all psychiatric units closed at the beginning of the Omicron surge as opposed to 38% during the Delta variant surge.^16^

During the Omicron surge in Chicago, Illinois, an outbreak occurred in the BHU at Northwestern Memorial Hospital (NMH), affecting both inpatients and HCWs. We established an outbreak investigation with 3 objectives: (1) to describe the outbreak and the COVID-19 mitigation measures implemented in regard to the BHU-specific physical infrastructure and standard of care, (2) to use molecular epidemiology and viral whole-genome sequencing (WGS) to elucidate SARS-CoV-2 transmission patterns within the BHU, and (3) to identify areas of improvement for future outbreak control plans in psychiatric care units. Here, we report the outcomes of that investigation, including the changes to infection prevention (IP) measures undertaken within the BHU to contain and minimize current and future outbreaks.

## Methods

### Outbreak investigation

This study describes the epidemiological and genomic characteristics of a COVID-19 outbreak in a BHU at NMH between December 2021 and January 2022. NMH is a 943-bed academic medical center in Chicago, Illinois, USA. The inpatient BHU is a 29-bed unit with 25 private rooms, 2 semi-private rooms, and several common rooms for group socialization. The BHU is a locked unit, with 24-hour monitoring by psychiatry HCWs and security. NMH experienced a surge of COVID-19 cases in mid-December 2021, coincident with the emergence of the SARS-CoV-2 Omicron variant in Chicago. The hospital IP team was notified of 2 inpatient COVID-19 cases in the BHU on December 16 and 20, 2021, which prompted a formal investigation. Point prevalence testing for all BHU inpatients and HCWs was initiated on December 21, 2021, in response to the outbreak investigation. Three additional individuals (1 inpatient and 2 HCWs) tested positive for SARS-CoV-2, prompting an official COVID-19 outbreak declaration. At the time, IP defined an outbreak as 3 or more individuals testing positive for COVID-19, who shared time, space, and/or healthcare providers. Thereafter, all patients and HCWs were screened weekly; patients and HCWs testing positive were excluded from subsequent testing. IP conducted weekly rounds on the BHU and performed daily surveillance. Additional IP measures included a temporary halt to group programming and communal meals. Outbreak resolution, defined as 1 week without a new positive COVID-19 patient or HCW, was declared on January 24, 2022. This was the only inpatient COVID-19 outbreak that occurred at NMH from December 2021 to January 2022.

### COVID-19 screening

COVID-19 screening for inpatients was performed using multiple polymerase chain reaction (PCR)-based diagnostic tests. COVID-19 screening for HCWs was performed using either the Alinity M or Roche-Cobas 8800 SARS-CoV-2 multiplex quality real-time reverse-transcriptase (qRT)-PCR test. All tests were conducted by the hospital’s Clinical Microbiology Laboratory and had Emergency Use Authorization from the Food and Drug Administration (FDA) at the time of use. Results were obtained and reported within 24 hours of sample collection. All isolates were collected via nasopharyngeal swabs in viral transport media.

### Research sample collection and viral load determination

Residual diagnostic specimens from individuals testing positive for SARS-CoV-2 in the Northwestern Medicine healthcare system were collected as part of an established biobank in the Center for Pathogen Genomics and Microbial Evolution at the Northwestern University Feinberg School of Medicine under study protocols STU00206850 and STU00212260. At the request of IP, specimens linked to the BHU outbreak were prioritized for viral WGS. Specimens were recovered from 27 of the 43 individuals involved in the outbreak investigation. Viral ribonucleic acid was extracted from each specimen using the QIAamp 96 Virus QIAcube HT Kit (Qiagen). The presence of SARS-CoV-2 was confirmed by qRT-PCR using the Centers for Disease Control and Prevention (CDC) 2019-nCoV RT-PCR Diagnostic Panel with the N1 and ribonucleases (RNase) P probes as previously described.^17^ Positive and negative controls for SARS-CoV-2 and RNase P were included in each qRT-PCR experiment alongside a no template control and standard curves for SARS-CoV-2 and RNase P.

### cDNA synthesis and viral genome amplification

Complementary DNA (cDNA) synthesis was performed with SuperScript IV First-Strand Synthesis Kit (Thermo) using random hexamer primers according to the manufacturer’s specifications. Direct amplification of the viral genome cDNA was performed in multiplexed PCR reactions to generate ∼400 base pair amplicons tiled across the genome as designed by the Artic Network (version 4.1 releases^18^). PCR amplification was carried out as previously described.^19^

### Sequencing library preparation and whole-genome sequencing

Sequencing library preparation of genome amplicon pools was performed using the SeqWell plexWell 384 kits per the manufacturer’s instructions. Pooled libraries were sequenced on the Illumina MiSeq using the V2 500 cycle kit. Sequencing reads were trimmed to remove adapters and low-quality sequences using Trimmomatic v0.36. Trimmed reads were aligned to the reference genome sequence of SARS-CoV-2 (accession MN908947.3) using bwa v0.7.15. Pileups were generated from the alignment using samtools v1.9, and the consensus sequence was determined using iVar v1.2.2 with a minimum depth of 10, a minimum base quality score of 20, and a consensus frequency threshold of 0 (ie, majority base as the consensus). Of the 27 recovered specimens, 4 failed to sequence to sufficient coverage, likely due to their relatively low viral loads (they all had N1 cycle threshold values above 30). A lineage designation was made for the remaining 23 SARS-CoV-2 consensus sequences using the PANGOLIN tool (Pango v4.2; data update: v1.18.1.1). All consensus sequences and lineage designations have been deposited to the GISAID repository (accession numbers available in Table S3 (online)).^20^

### Phylogenetic analysis

Genome sequences were aligned using MAFFT v7.453 software,^21^ and maximum likelihood (ML) phylogenies were inferred with IQ-Tree2 v2.0.5^22^ using its ModelFinder function^23^ before each analysis to estimate the best-fit nucleotide substitution model for each data set using Bayesian information criterion. We assessed the tree topology for each phylogeny both with the Shimodaira–Hasegawa approximate likelihood-ratio test (SH-aLRT)^24^ and with ultrafast bootstrap^25^ with 1,000 replicates each. TreeTime v0.7.6^26^ was used for the assessment of root-to-tip correlation, the estimation of time-scaled phylogenies, and the ancestral reconstruction of most likely sequences of internal nodes of the tree. TreeTime was run using an autocorrelated molecular clock under a skyline coalescent tree prior. We used the sampling dates of the sequences to estimate the evolutionary rates and determine the best rooting of the tree using root-to-tip regression with the least-squares method. For Cook County ML phylogenies, we downloaded from GISAID 1,023 SARS-CoV-2 sequences identified as Omicron clades sampled in Cook County, Illinois, between December 1, 2021, and January 31, 2022. We inferred ML phylogeny using MAFFT and IQ-Tree2 with these sequences and the ones obtained for this study to confirm the clustering observed. Metadata associated with these 1,043 sequences are available on GISAID at https://doi.org/10.55876/gis8.230902od.

### Phylogenetic clustering

To assess transmission clusters, we used 2 complementary methods. First, we defined transmission clusters as sequences that cluster in the ML phylogeny with strong statistical support using SH-aLRT >70% and bootstrap >80%. Second, we defined transmission clusters as using the Max Clade method in the TreeCluster tool^27^ with an applied maximum pairwise distance of less than 5 × 10^–6^ substitutions per base pair within a cluster. The transmission clusters indicated here were supported by both methods.

## Results

### COVID-19 mitigation strategy in the BHU

**Figure 1.**
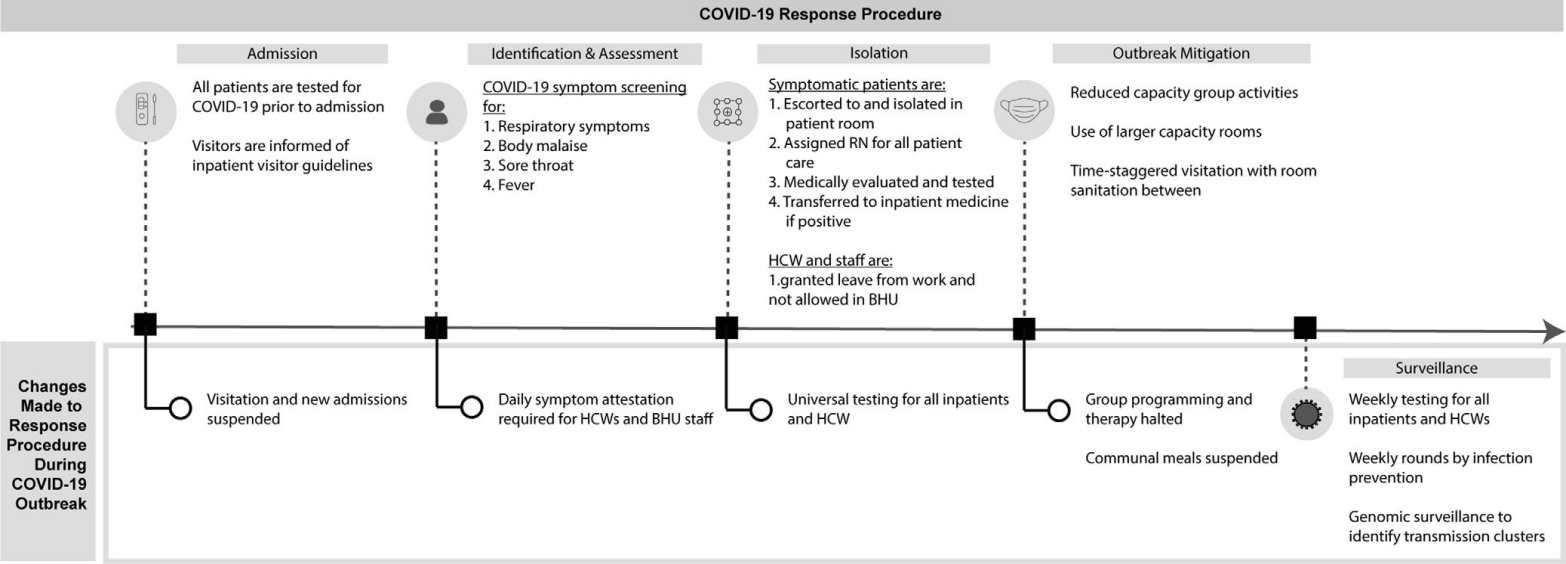
COVID-19 response procedure in the BHU at NMH. The NMH infection prevention team implemented a response plan at the start of the COVID-19 pandemic as summarized before the COVID-19 outbreak (top). Additional measures implemented during the December 2021–January 2022 COVID-19 outbreak in the BHU, including the implementation of molecular surveillance, are included (bottom). *Note*: BHU, behavioral health unit; COVID-19, coronavirus disease 2019; NMH, Northwestern Memorial Hospital.

At the start of the COVID-19 pandemic in March 2020, the IP team crafted a series of COVID-19 mitigation policies with recommendations for patients, staff, and visitors to the BHU (Figure 1). The screening and response procedure was designed to minimize points of potential transmission beginning at admission when all patients were tested for SARS-CoV-2 before BHU admission. Patients were additionally tested before any procedures requiring anesthesia, such as electroconvulsive therapy (ECT), or deemed likely to produce aerosols, and before transfer to another psychiatric facility. If an inpatient developed symptoms consistent with COVID-19 (respiratory symptoms, malaise, sore throat, and/or fever), the patient was isolated in their room, medically evaluated, and tested. If the patient tested positive for SARS-CoV-2, they were transferred to a different medical unit within the hospital with the capacity to accommodate COVID-19 patients. Throughout this period, the BHU required universal masking with surgical grade masks or higher for all patient–staff interactions, visitation, and group activity sessions. HCW and staff were additionally required to wear goggles, gloves, and biosafety gowns per standard precautions. N95 respirators were only required for HCWs during any aerosol-producing procedures, such as continuous positive airway pressure therapy and ECT, or for staff interacting with patients in isolation due either to symptoms of COVID-19 or positive COVID-19 testing prior to transfer out of the BHU to a medical unit for evaluation and treatment.

Additional measures were taken to accommodate social distancing and visitation without compromising the essential socialization practices of a BHU. Group activities were moved to larger capacity rooms, and participant capacity was decreased from 27 to 13. Smaller, enclosed areas were either cordoned off or subject to reduced capacity guidelines. Visitors were screened for symptoms by hospital staff before admission to the BHU and were required to follow all posted NMH and BHU-specific safety guidelines. Visitation was limited to specified 45-minute intervals, and visitation rooms were sanitized between intervals. A virtual platform option was added as an alternative to in-person visitation.

### COVID-19 outbreak description and mitigation strategy

NMH and the greater Chicago area experienced a surge of COVID-19 cases in mid-December 2021, coincident with the emergence of the SARS-CoV-2 Omicron variant in the region. The NMH IP team was notified of 2 BHU inpatients who tested positive for SARS-CoV-2 on December 16 and 20, 2021, which prompted a formal outbreak investigation. Point prevalence testing for all BHU inpatients and HCWs on December 21, 2021, uncovered 3 additional cases (1 inpatient and 2 HCWs) resulting in an official outbreak declaration. Per the COVID-19 response plan, all patients testing positive for COVID-19 were transferred to another inpatient unit at NMH regardless of symptoms.

At the onset of the outbreak declaration, the IP team modified the COVID-19 screening and response plan. Four successive rounds of mitigation policies were implemented on December 21, 23, and 28, 2021, and on January 4, 2022, to expand on the existing response plan (Figure 1, Table S1 (online)). All visitation, large group activities, group therapy sessions, and communal meals were suspended and replaced with individualized sessions. The proper use of PPE was reinforced, and the use of N95 respirators instead of surgical grade masks was mandated for HCWs and staff in all patient interactions. PCR-based COVID-19 diagnostic tests were administered to all inpatients, HCWs, and staff weekly. HCW and staff were further required to attest to the lack of symptoms before entering the BHU daily. IP conducted weekly rounds on the BHU and performed daily surveillance of COVID-19-related symptoms.

**Figure 2.**
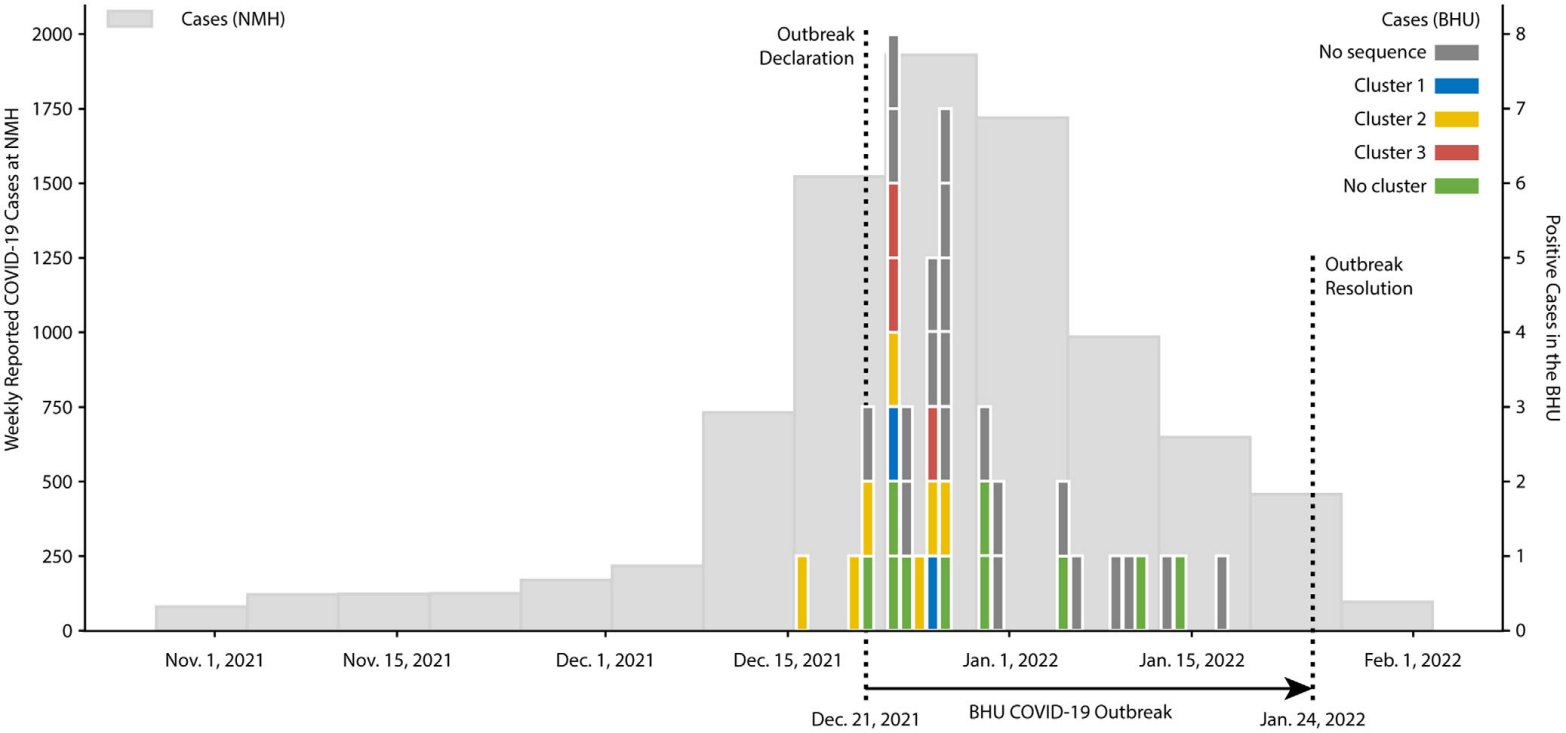
COVID-19 outbreak time line in Northwestern Memorial Hospital and the BHU. Weekly counts of COVID-19 cases reported at Northwestern Memorial Hospital between the weeks of November 1, 2021, and February 1, 2022, are illustrated in light gray (left axis). Daily counts of COVID-19 cases in the BHU between the weeks of December 15, 2021, and January 15, 2022, are overlaid and colored by outbreak cluster (right axis). Hashed lines indicate the start and end of the BHU outbreak. *Note*: BHU, behavioral health unit; COVID-19, coronavirus disease 2019

The outbreak lasted nearly 6 weeks before being declared resolved on January 24, 2022, after 7 days with no new cases being detected. A total of 43 individuals (8 inpatients (18.2%) and 35 HCWs (81.8%)) tested positive over this time frame (Table 1, Figure 2). The staff included in the outbreak were security personnel, HCWs, ancillary staff, and students, who frequented the BHU with varying levels of staff-to-patient interaction. This was the only inpatient COVID-19 outbreak that occurred at NMH between December 2021 and January 2022. Following outbreak resolution, the additional mitigation measures were halted, though the response plan was subsequently amended to limit the size of group therapy sessions and to require the use of N95 respirators by all HCWs and ancillary staff during patient–staff interactions.

**Table 1.** Patient demographics of individuals involved in the COVID-19 outbreak in the BHU

Variable	Patient, n = 8^a^	HCW = 35^a^
**Age**	40 (31, 52)	41 (30, 51)
**Sex**		
Male	5 (63%)	19 (54%)
Female	3 (38%)	16 (46%)
**Race**		
Black or African American	2 (25%)	14 (40%)
White	4 (50%)	8 (23%)
Unknown	1 (13%)	9 (26%)
Asian	1 (13%)	4 (11%)
**Ethnicity**		
Not Hispanic or Latino	8 (100%)	27 (77%)
Hispanic or Latino	0 (0%)	4 (11%)
Unknown	0 (0%)	4 (11%)
**BMI**	23 (21,26)	27 (24,32)
**Vaccination**		
Vaccinated	3 (38%)	16 (45.7%)
Boosted	2 (25%)	10 (28.6%)
Unknown	1 (13%)	7 (20%)
Partially vaccinated	0 (0%)	2 (5.7%)
Unvaccinated	2 (25%)	0 (0%)
^a^Median (IQR); n (%)		

Note. COVID-19, coronavirus disease 2019; BHU, behavioral health unit; BMI, body mass index; IQR, interquartile range; HCW, healthcare worker.

a Median (IQR) for continuous variables; n (%) for categorical variables; HCW and BHU patients are stratified.

### Molecular epidemiology

SARS-CoV-2 WGS was performed on residual diagnostic specimens from COVID-19 testing. Of the 43 individuals involved in the outbreak, residual diagnostic specimens were recovered from 27 (62.7%). Of these, 23 (85.2%) had sufficient viral load and were of sufficient quality to yield a full-length WGS, including from all 8 inpatients and 15 HCWs (Table S2 and S3 (online)).^20^ Of the sequenced isolates, 1 was a Delta variant, and the other 22 were Omicron variants.

**Figure 3.**
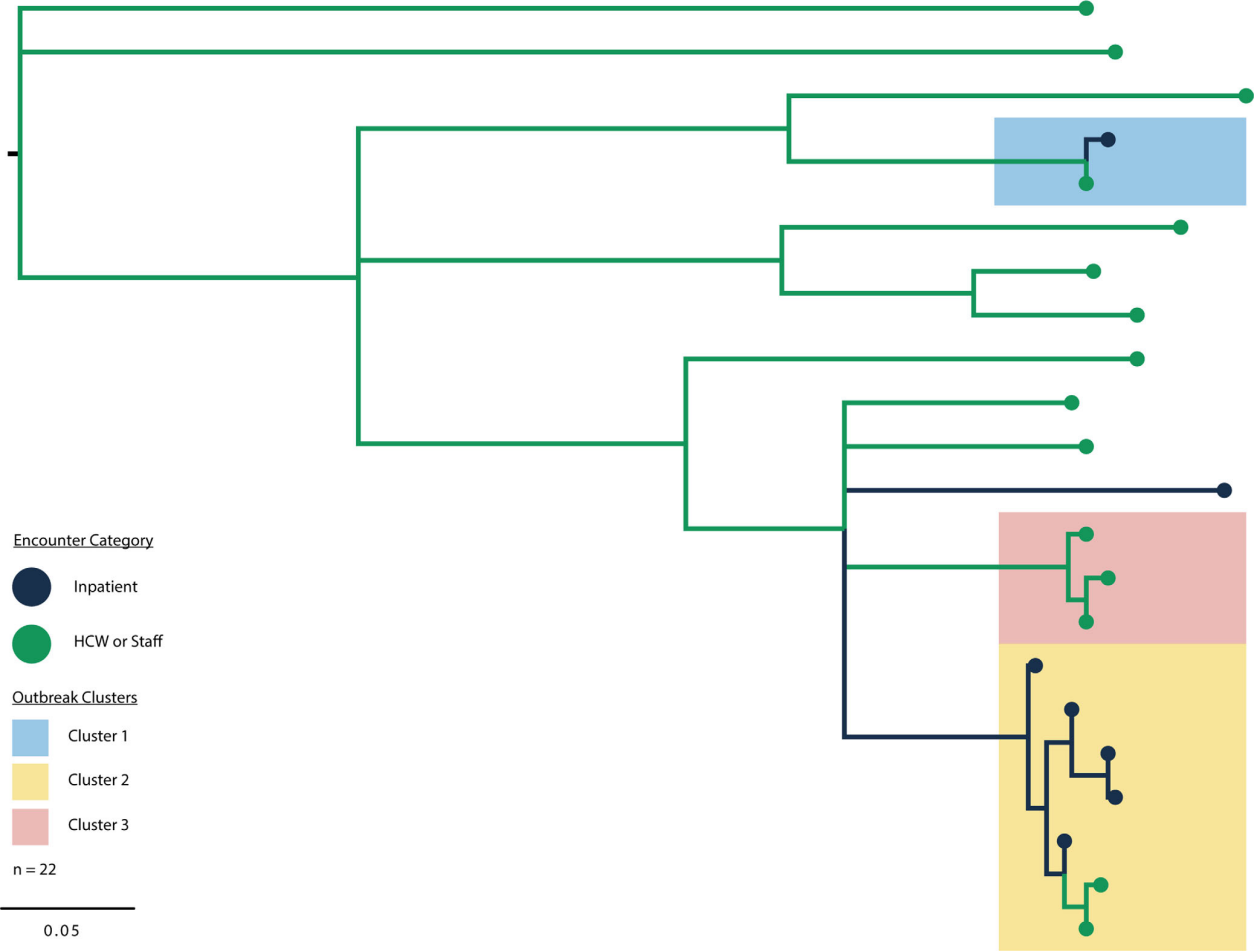
SARS-CoV-2 phylogenetic outbreak clusters. Phylogenetic tree of SARS-CoV-2 Omicron whole-genome sequences from the BHU outbreak (n = 22). The single Delta sequence was excluded for clarity. Healthcare workers (HCWs) and staff specimens are annotated as green branches and taxa, although patients are annotated in black. Probable clusters based on phylogenetic similarity are highlighted as Cluster 1 (blue), Cluster 2 (yellow), and Cluster 3 (red). *Note*: BHU, behavioral health unit; SARS-CoV-2, severe acute respiratory coronavirus virus 2

To determine if the Omicron isolates represented independent introductions versus 1 or more transmission clusters, a ML phylogenetic tree was constructed from the 22 Omicron isolates (Figure 3). This analysis revealed 3 distinct outbreak clusters with high statistical support (refer to Methods: ‘Phylogenetic clustering’): Cluster 1 was comprised of a transmission pair involving 1 HCW and 1 inpatient, Cluster 2 contained 5 inpatients and 2 HCWs, and Cluster 3 involved no inpatients and 3 HCWs and had a higher intra-cluster diversity than the other 2 clusters. When considering the time of infection, Cluster 2 appeared to be driven by an initial inpatient infection, likely originating from a visitor, while Clusters 1 and 3 likely originated from HCW infections. The 10 other Omicron cases (1 inpatient and 9 HCWs) and the Delta case appear to be unrelated, independent infections based on available data.

The peak of the outbreak in the BHU coincided with a surge of COVID-19 cases reported and/or detected at NMH (Figure 2). Given the high community prevalence at this time and the relatively low viral genetic diversity of the Omicron variant during its initial expansion, it is possible that similar viral sequences may not be representative of a transmission cluster but rather represent independent infection events. To assess this possibility, the phylogenetic analysis was expanded to include 1,023 SARS-CoV-2 Omicron sequences sampled in Cook County, Illinois, over this same time frame between December 1, 2021, and January 31, 2022. (Figure S4 (online)). Although the BHU specimens in Clusters 1 and 2 remained tightly associated (aLRT >90% and bootstrap >90%), again suggesting true transmission clusters, the specimens in Cluster 3 did not cluster independently and showed no significant genetic diversification in comparison to several other isolates collected in the county over the same time. This together with their higher intra-cluster diversity suggests that Cluster 3 may either represent a broader transmission cluster or may be the result of independent infections with genetically similar viruses.

**Figure 4.**
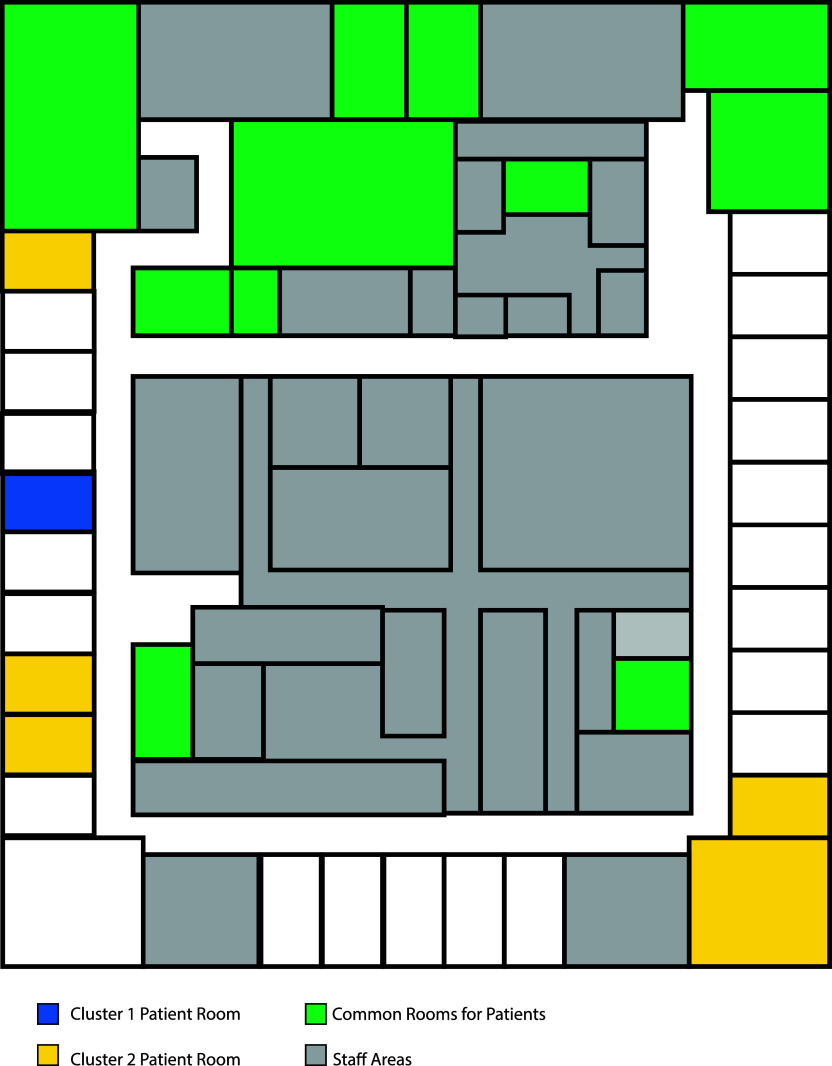
BHU floorplan. The map represents an overhead view of the BHU floorplan with staff areas highlighted in gray and common rooms for group activities highlighted in green. Inpatient rooms in which patients involved in one of the suspected transmission clusters resided are highlighted with Cluster 1 in blue and Cluster 2 in yellow. Cluster 3 involved no inpatients and so is not represented. *Note*: BHU, behavioral health unit.

We further expanded our analysis to understand whether the transmission of these clusters was influenced by interactions within specific areas of the BHU. The location of patient rooms involved in the outbreak had no clear association with the transmission cluster (Figure 4). Consequently, infections within the BHU were likely a result of interactions in communal areas, supporting early mitigation steps taken during the outbreak to suspend the use of these rooms for group activities. Overall, this outbreak investigation suggested multiple, independent introductions of the virus to the BHU with at least 2 and possibly 3 transmission clusters (Table 2).

**Table 2.** Epidemiological conclusions of identified SARS-CoV-2 clusters in the BHU outbreak

Cluster	# of HCWs	# of patients	Epidemiological findings
1	1	1	A transmission pair where an HCW spread SARS-CoV-2 to an inpatient. The HCW is suspected to have acquired SARS-CoV-2 from a community-based transmission
2	2	5	An inpatient likely acquired SARS-CoV-2 from a visitor, potentially due to a lack of adherence to COVID-19 guidelines. This likely spread to additional inpatients and HCWs through group interactions in communal settings
3	3	0	A potential transmission cluster of HCWs, though low genetic variability of the virus at this time prevented differentiation of nosocomial versus community-based acquisition in this cluster

Note. SARS-CoV-2, severe acute respiratory coronavirus virus 2; BHU, behavioral health unit; HCWs, healthcare workers; COVID-19, coronavirus disease 2019.

## Discussion

The impact of COVID-19 on the inpatient BHUs highlights the challenges in balancing outbreak mitigation measures with optimal patient care. The outbreak highlighted the need for an effective and rapid response to curb any future outbreaks in the BHU.^28^ SARS-CoV-2 can be transmitted rapidly within congregate residential units, as seen early in the pandemic in nursing and other group homes^29,30^ and as reflected in later outbreaks in hospital BHUs.^13^ BHUs represent a unique hospital setting where most of a patient’s time is spent in group activities, social interaction, and visitation.^11^ The need for patient socialization conflicts with several COVID-19 mitigation measures, including social distancing and quarantine. Therefore, BHU-specific measures necessitate a flexible response plan that relies on additional tools including surveillance and self-reporting.

Rapid identification of cases, coordination with IP, and implementation/enforcement of iterative mitigation measures helped to limit the duration of the described BHU outbreak to 6 weeks. Clinical and epidemiological investigations identified a total of 43 cases, including 8 inpatients and 35 HCWs and staff. Molecular epidemiological follow-up on 23 specimens using SARS-CoV-2 WGS found that 12 of the cases belonged to 3 distinct transmission clusters, although 11 could be attributed to community-acquired infections. Community transmission of the Omicron variant was high during the time of the study, which likely contributed to the high number of introductions observed over this period. Many of the independent introductions were linked to HCWs and staff, most of whom acquired SARS-CoV-2 from the community as opposed to from nosocomial infection. Several inpatient cases were linked to visitors, including 1 that resulted in a transmission cluster on the unit. Additional mitigation measures introduced during the outbreak, such as enhanced PPE requirements for HCWs and staff, increased testing, and a suspension of visitation, likely contributed to the containment of the outbreak.

Prior surveillance studies in both healthcare and community settings illustrate the utility of WGS to identify infection transmission patterns and translate findings to new policies. SARS-CoV-2 WGS studies in academic settings have been useful in re-evaluating mitigation interventions,^32^ although similar studies in healthcare settings have resulted in the reinforcement of masking policies^33^ or the re-evaluation of communal space usage.^34^ However, a recent meta-analysis of reports on the use of WGS in healthcare settings found that only 18% used these data to directly inform IP policy or implementation.^35^ Our study contributes to an increasing body of literature that suggests a broader use of WGS in hospital epidemiological investigations can effectively inform mitigation policy while maintaining standards of care.

There were some limitations to the study. The molecular epidemiological investigation relied on the collection of residual diagnostic specimens from individuals tested at NMH, of which only 62.8% were recoverable. Unrecovered specimens reflected the use of testing facilities outside of the Northwestern Medicine (NM) system or the use of a clinical diagnostic platform that does not yield a residual sample. A lack of sequencing information may have resulted in an underestimation of nosocomial transmission events. Additionally, the low genetic variability of the SARS-CoV-2 Omicron variant at the time of the study complicates the interpretation of some clusters where the genetic similarity between the cases may have been coincidental due to the high prevalence of the viral genotype in the surrounding community.

To avoid potential outbreaks in the future, BHUs and other clinical care units may consider adopting additional guidelines during times of high community transmission. Measures such as universal masking, social distancing, screening of visitors, and weekly point prevalence testing have the potential to break the chain of transmission and significantly decrease the likelihood of an outbreak.^35^ Flexible response plans that employ mitigation measures as needed depending on the circumstance in the BHU, hospital, and community are likely to provide the most protection with the least detriment to patient care. Mitigation plan development should involve not only staff in the BHU but also include IP teams and molecular and diagnostic testing labs as the burden of outbreak management is shared. As hospital and clinical care units relax their mitigation policies in response to the end of the global COVID-19 pandemic,^36^ these lessons may guide BHUs and other healthcare systems as they respond to nosocomial transmissions of infectious diseases with outbreak potential such as SARS-CoV-2 or influenza, for example, in the future.

## Supporting information

Rios-Guzman et al. supplementary material Rios-Guzman et al. supplementary material

## Supplementary material

For supplementary material accompanying this paper visit http://doi.org/10.1017/ash.2024.40. click here to view supplementary material
