# Supplementary material for: COVID-19 outbreak and genomic investigation in an inpatient behavioral health unit
Source: Antimicrob Steward Healthc Epidemiol. 2024 Apr 29;4(1):e62. doi: 10.1017/ash.2024.40 (PMC11062797; doi:10.1017/ash.2024.40)
Supplement: Rios-Guzman et al. supplementary material [file S2732494X24000408sup001.docx]

| **Study Period** | **Effective Dates** | **COVID-19 Risk Mitigation Policy** |
| --- | --- | --- |
| Pre-Outbreak Period | before December 21, 2021 | Patients screened for COVID-19 before admission to the BHU. Universal masking with additional PPE required for HCW and staff, including goggles, gowns, and gloves per standard precautions. Employees were instructed to self-screen for fever and other symptoms of COVID-19 daily and attest to a lack of symptoms prior to work via an electronic application. Patients were monitored for symptoms. If a patient became symptomatic, they were isolated in their room, tested, and transferred to another inpatient unit if positive. Group activities were held in larger rooms with smaller numbers of individuals. Visitation was timed and scheduled with sanitation of rooms between visits. |
| Mitigation Set 1 | December 21, 2021 | Visitation and admission of new patients were suspended. All patients, healthcare workers, and personnel who were on the floor for 2 weeks prior to the first patient testing positive were screened for COVID-19 by PCR-based diagnostic test. Weekly testing was enforced thereafter. Large group activities and group therapy sessions were suspended. Communal meals were suspended, and patients had meals served in their rooms. Social distancing enforced for small group interactions. |
| Mitigation Set 2 | December 23, 2021 | Weekly rounds for infection prevention were started. |
| Mitigation Set 3 | December 28, 2021 | PPE requirements for HCW and staff were reemphasized. N95 masks and eye protection were supplied to all staff. |
| Mitigation Set 4 | January 4, 2022 | The comfort therapy room went to single-patient use. |
| Post-Outbreak Period | after January 24, 2022 | Group therapy sessions remained at a lower capacity in larger rooms. The comfort room was re-opened with new policies including surface disinfection and 35-minute wait times between uses for aerosols to disperse. N95 masks were required for HCWs and staff. |

**Supplementary Table 1 | Risk mitigation strategies implemented before, during, and after the BHU outbreak.**

**
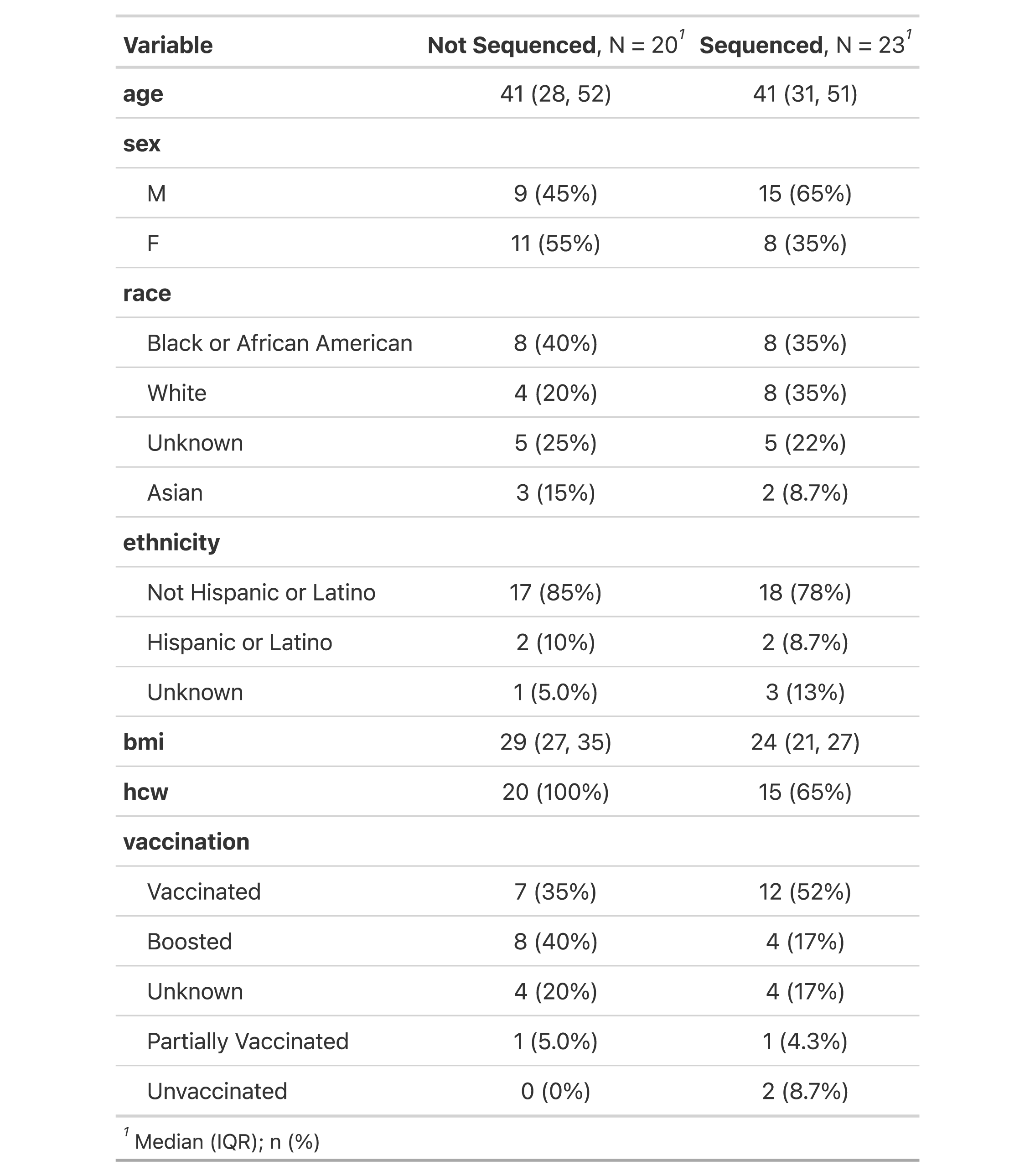
**

**Supplementary Table 2 | Patient demographics of individuals involved in the COVID-19 outbreak in the BHU.** *^1^*Median (IQR) for Continuous variables; n (%) for Categorical variables; COVID-19 encounters with and without sequencing information are stratified.

**
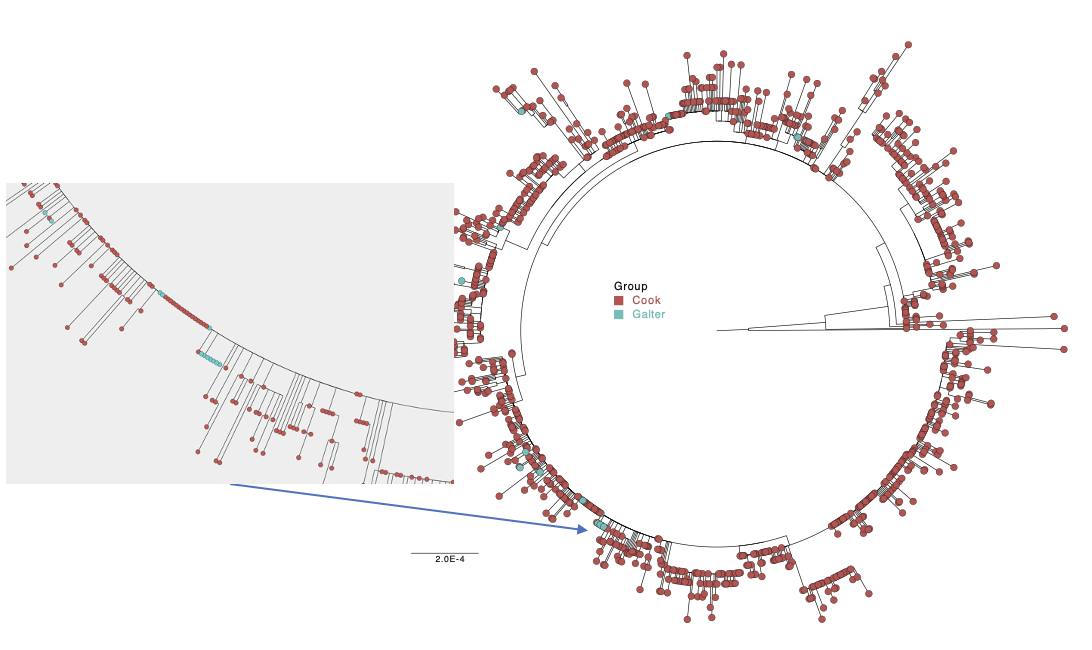
**

**Supplementary Figure 1 | Phylogenetic Analysis of SARS-CoV-2 Isolates Sampled in Cook County, Chicago, Illinois.** Maximum likelihood (ML) phylogenetic analysis of Omicron SARS-CoV-2 whole genome sequences from NMH BHU (blue, n =22) and Cook County, Illinois (red, n = 1042) collected from December 1, 2021 – January 31, 2022. Zoomed in box and arrow indicate the genetic similarity of 3 BHU genomes to other isolates sequenced in Cook County.

| GISAID ID | Accession ID | Lineage | Clade |
| --- | --- | --- | --- |
| hCoV-19/USA/IL-NM-14478/2021 | EPI_ISL_9912566 | BA.1.20 | GRA |
| hCoV-19/USA/IL-NM-15440/2021 | EPI_ISL_9912568 | BA.1.20 | GRA |
| hCoV-19/USA/IL-NM-15492/2021 | EPI_ISL_9912569 | BA.1.20 | GRA |
| hCoV-19/USA/IL-NM-15780/2021 | EPI_ISL_9912570 | BA.1 | GRA |
| hCoV-19/USA/IN-NM-16224/2021 | EPI_ISL_9912571 | BA.1.20 | GRA |
| hCoV-19/USA/IL-NM-16225/2021 | EPI_ISL_9912572 | BA.1 | GRA |
| hCoV-19/USA/IL-NM-16233/2021 | EPI_ISL_9912573 | BA.1.20 | GRA |
| hCoV-19/USA/IL-NM-16368/2021 | EPI_ISL_9912574 | BA.1.15 | GRA |
| hCoV-19/USA/IL-NM-16625/2021 | EPI_ISL_9912575 | BA.1.20 | GRA |
| hCoV-19/USA/IL-NM-18298/2022 | EPI_ISL_9912577 | BA.1 | GRA |
| hCoV-19/USA/IL-NM-19936/2021 | EPI_ISL_9912671 | BA.1.20 | GRA |
| hCoV-19/USA/IL-NM-19937/2021 | EPI_ISL_9912673 | BA.1.1.14 | GRA |
| hCoV-19/USA/IL-NM-19938/2021 | EPI_ISL_9912676 | BA.1.20 | GRA |
| hCoV-19/USA/IL-NM-19939/2021 | EPI_ISL_9912678 | BA.1 | GRA |
| hCoV-19/USA/IN-NM-19940/2021 | EPI_ISL_9912680 | BA.1.18 | GRA |
| hCoV-19/USA/IL-NM-19942/2021 | EPI_ISL_9912686 | BA.1.20 | GRA |
| hCoV-19/USA/IL-NM-19943/2021 | EPI_ISL_9912688 | AY.92 | GK |
| hCoV-19/USA/IL-NM-19944/2021 | EPI_ISL_9912690 | BA.1.20 | GRA |
| hCoV-19/USA/IL-NM-19945/2021 | EPI_ISL_9912692 | BA.1.20 | GRA |
| hCoV-19/USA/IL-NM-19946/2021 | EPI_ISL_9912694 | BA.1.20 | GRA |
| hCoV-19/USA/IL-NM-20051/2022 | EPI_ISL_9912696 | BA.1.20 | GRA |
| hCoV-19/USA/IL-NM-20565/2021 | EPI_ISL_9912698 | BA.1.15 | GRA |
| hCoV-19/USA/IL-NM-20657/2022 | EPI_ISL_9912700 | BA.1.15 | GRA |

**Supplementary Table 3 | GISAID Information for Specimens Sequenced in this study.**

**
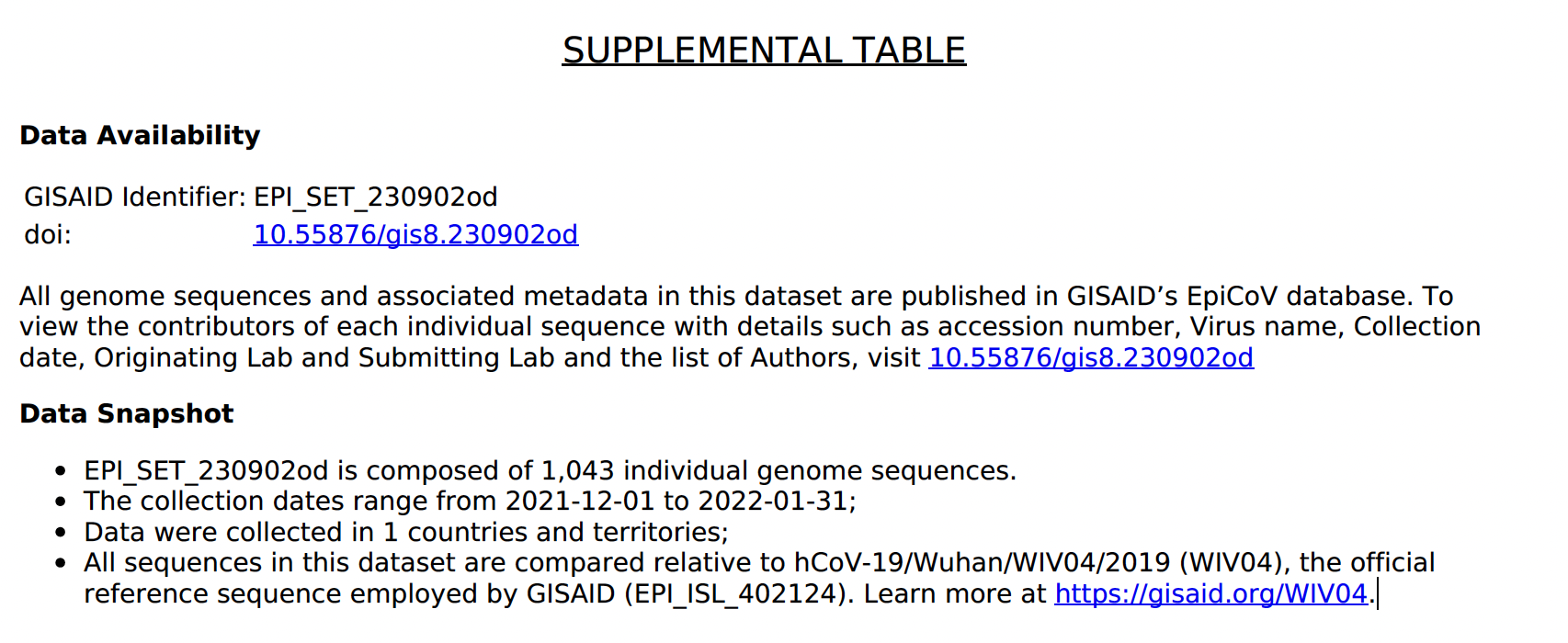
**

**Supplementary Table 4 | GISAID Information for Specimens Sequenced in this study and Cook County Genomes in Supplementary Figure 1.**
